# Supplementary material for: Alchemically-glazed plasmonic nanocavities using atomic layer metals: controllably synergizing catalysis and plasmonics
Source: Nat Commun. 2025 Apr 9;16:3370. doi: 10.1038/s41467-025-58578-9 (PMC11982554; doi:10.1038/s41467-025-58578-9)
Supplement: Supplementary file 1 — Supplementary Information [file 41467_2025_58578_MOESM1_ESM.pdf]

Alchemically-glazed plasmonic nanocavities using atomic layer metals: controllably synergizing catalysis and plasmonics

Shu Hu[1,2], Eric S. A. Goerlitzer[2], Qianqi Lin[2], Bart De Nijs[2], Vyacheslav M. Silkin[3,4], Jeremy J. Baumberg[2*]

[1] Department of Physics, Xiamen University, Xiamen, 361005, China
[2] Nanophotonics Centre, Dept. of Physics, Cavendish Laboratory, University of Cambridge, Cambridge, CB3 0HE, UK
[3] Donostia International Physics Center, P. de Manuel Lardizabal 4, 20018 San Sebastián/Donostia, Basque Country, Spain
[4] Centro de Física de Materiales, Centro Mixto CSIC-UPV/EHU, P. de Manuel Lardizabal, 5, 20018 San Sebastián/Donostia, Basque Country, Spain
* e-mail: jjb12@cam.ac.uk

Contents of Supplementary Information:

Supplementary Figure 1: AFM characterization of a template stripped Au film.
Supplementary Figure 2: Monolayer Pd deposition processes.
Supplementary Figure 3, 4: Characterization of monolayer Pd by synchrotron X-ray photoelectron spectroscopy ,electrochemistry and Raman spectroscopy.
Supplementary Figure 5: Photocurrent from NPoMs during electrochemical oxidation of ethanol.
Supplementary Figure 6: Photocatalytic current comparison of NPoMs and antenna reactors.
Supplementary Figure 7: Dark field spectra statistics of individual Pd-glazed NPoMs (biphenyl thiol SAM).
Supplementary Figure 8: Pd coverage dependent cavity mode wavelength and intensity of NPoMs.
Supplementary Figure 9, 10: Monolayer Pd induced cavity mode change of NPoMs with BPDT and PDI SAM.
Supplementary Figure 11: Monolayer Pd induced cavity mode wavelength change of NDoMs.
Supplementary Figure 12: The frequency distribution of Au-S vibration of various NPoM constructs.
Supplementary Figure 13: DFT simulations of biphenyl thiol molecule adsorbed on Au and Pd.
Supplementary Figure 14: Comparison of SERS intensity of NPoMs and UPoMs with and without Pd glazing.
Supplementary Figure 15: Comparison of SERS intensity of NPoMs and Pd-glazed NPoMs with a BPDT SAM.
Supplementary Figure 16: SERS characterization of Ag, Cu, Pd and Pt glazed NPoMs.
Supplementary Figure 17: Photocatalytic current measurement of hydrogen evolution reaction and CO_2 reduction on Pt and Cu glazed NPoMs.
Supplementary Table 1: Additional literature for alchemical capabilities.
Supplementary Figure 18: Schematic of electrochemical cell.
Supplementary Figure 19: SEM image of NPoMs on Au substrate.

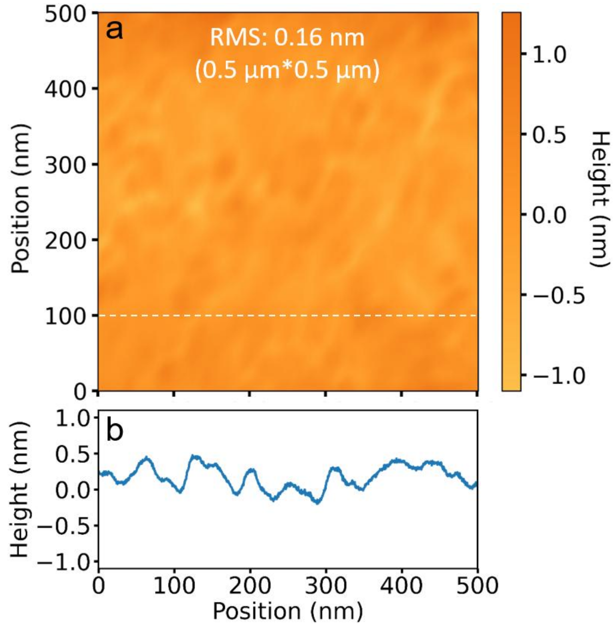

Supplementary Figure 1. AFM (**a**) image and (**b**) line scan (dashed line in **a**) of a template stripped Au film.

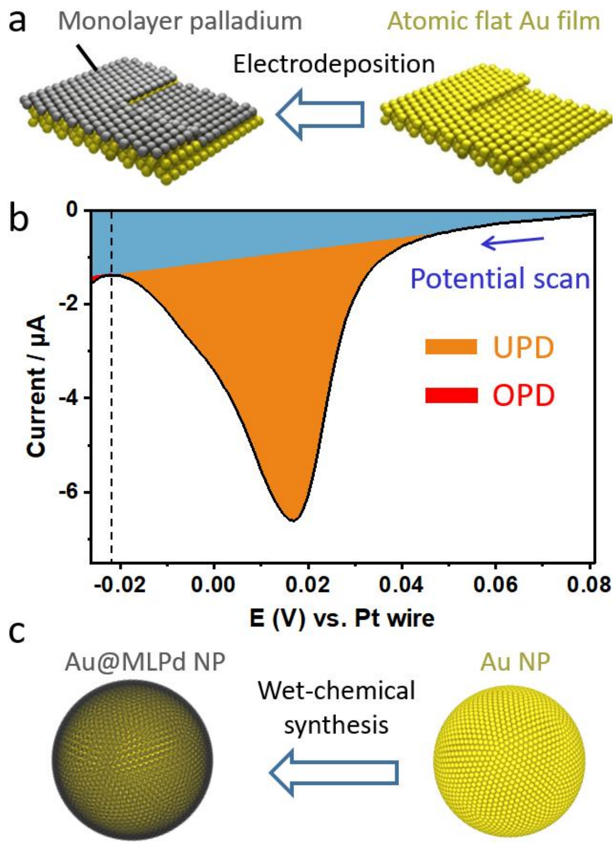

Supplementary Figure 2. Schematic of monolayer Pd deposition on (**a**) Au substrate and (**c**) Au nanoparticle. (**b**) Linear sweep voltammetry curve of electrochemical underpotential and overpotential deposition of Pd on a template stripped Au film.

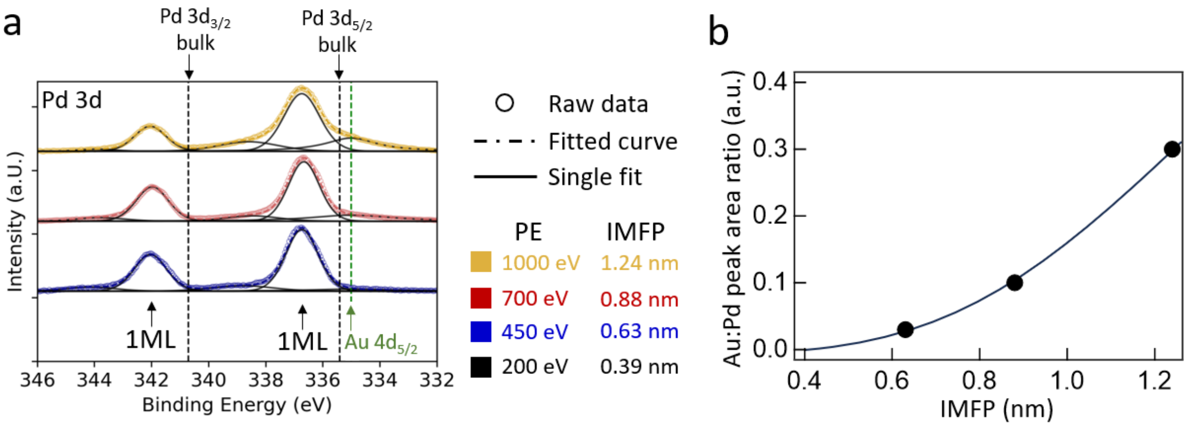

Supplementary Figure 3. a, Synchrotron X-ray photoelectron spectroscopy measurements of the monolayer Pd on Au substrate for different photon energy (PE) illumination. Assigned bands of Pd 3d and Au 4d are shown dashed. Colors label the different PE and inelastic mean free path (IMFP). **b,** IMFP dependent variation of Au:Pd peak area ratio for ~336 eV bands.

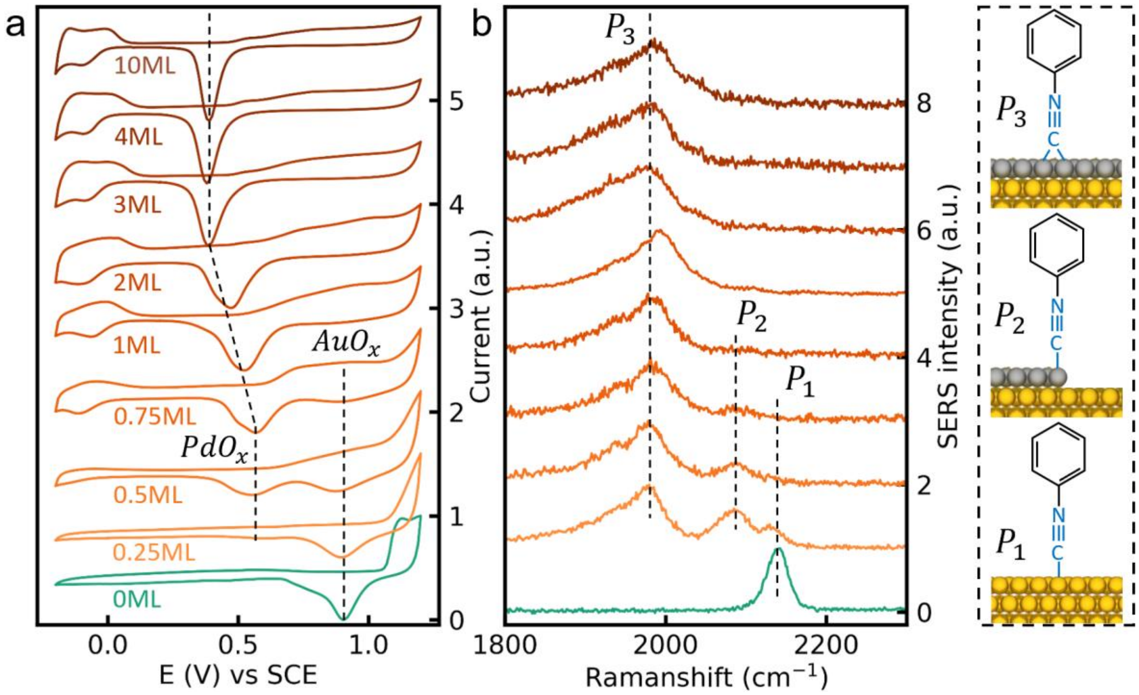

Supplementary Figure 4. a, Cyclic voltammetry curves of the Au (green) and Au@Pd (orange to brown) NPoMs with thickness of Pd increasing from submonolayer to 10 monolayers. Electrolyte is 0.1M H_2SO_4. Dashed lines highlight the potential of AuO_x and PdO_x reduction. **b**, Surface enhanced Raman scattering (SERS) spectra of the corresponding nanoparticles in (**a**) for phenyl isocyanide molecules absorbed on the surface as a probe. Dashed lines and schematics (right) show the different vibration energies when N≡C triple bonds adsorb on regions with different surface atomic species.[1,2]

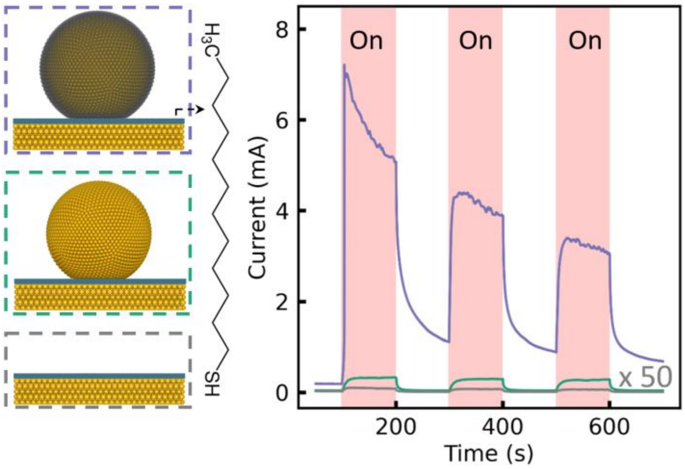

Supplementary Figure 5. Schematics (left) of different substrates and their corresponding photocurrent (right) under light illumination (600-900nm) of a same power of 0.3W·cm^{-2}. The electrolyte is 0.1M NaOH and 0.1M C_2H_5OH. Potential is held at -0.2V.

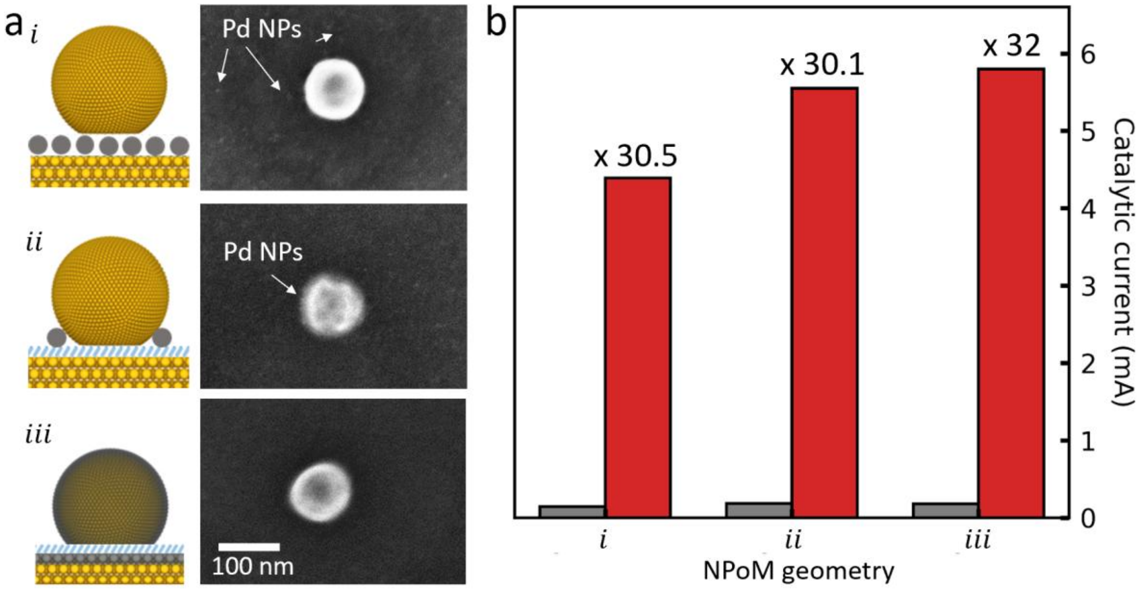

Supplementary Figure 6. **a**, Different catalytic nanocavity geometries where (i) 6nm Pd nanoparticles (NPs) are assembled inside the NPoM metallic gap, (ii) Pd NPs surround the NPoM (1-DDT SAM spacer in blue) and (iii) a Pd-glazed NPoM (1-DDT SAM spacer). **b**, Catalytic photocurrent from ethanol oxidation in different geometries of (**a**). Red is light on (0.3W·cm^{-2}), grey is light off.

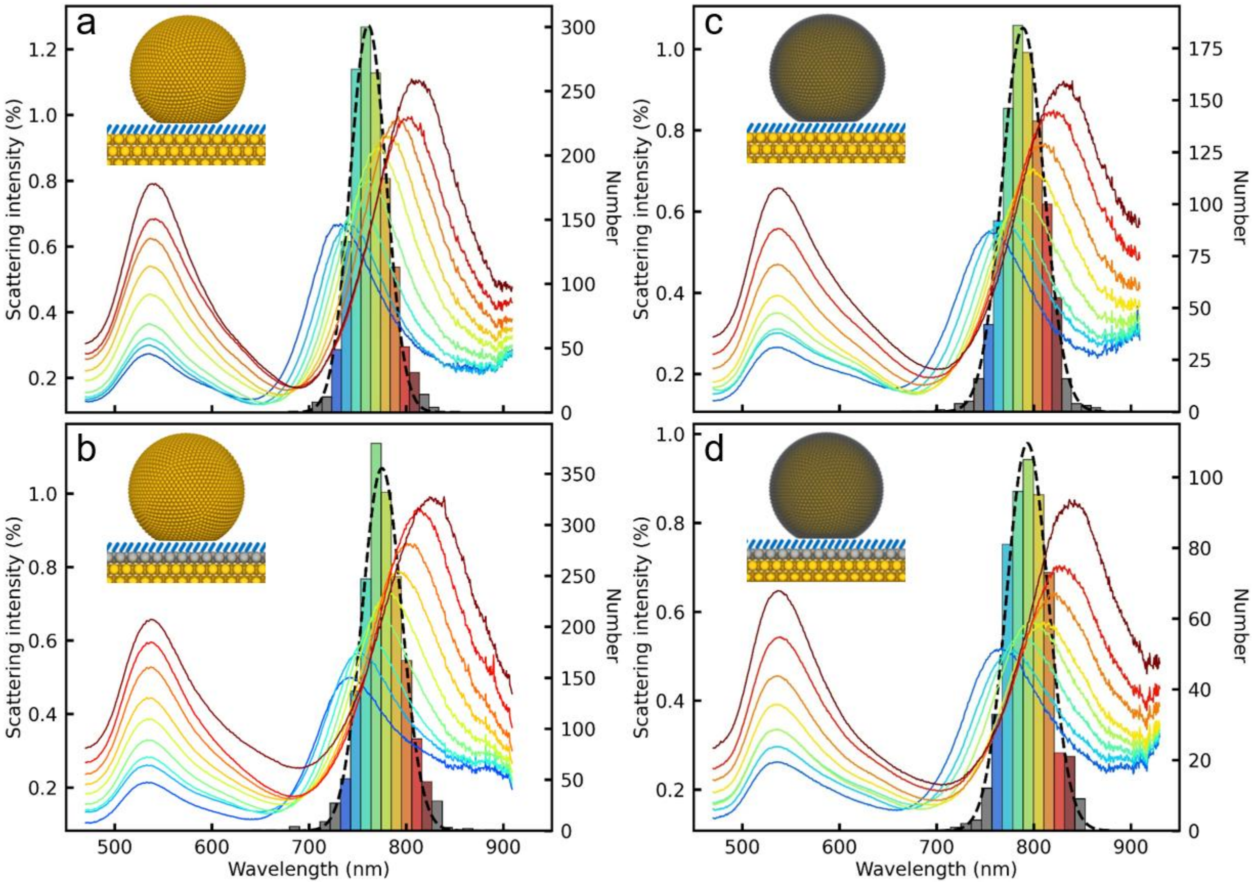

Supplementary Figure 7. Histograms of the (10) mode wavelengths and average spectra from each correspondingly coloured bin of NPoMs, (**a**) without Pd, (**b**) with monolayer Pd on bottom Au film, (**c**) Pd ML on top Au nanoparticle only, and (**d**) Pd ML on both top and bottom.

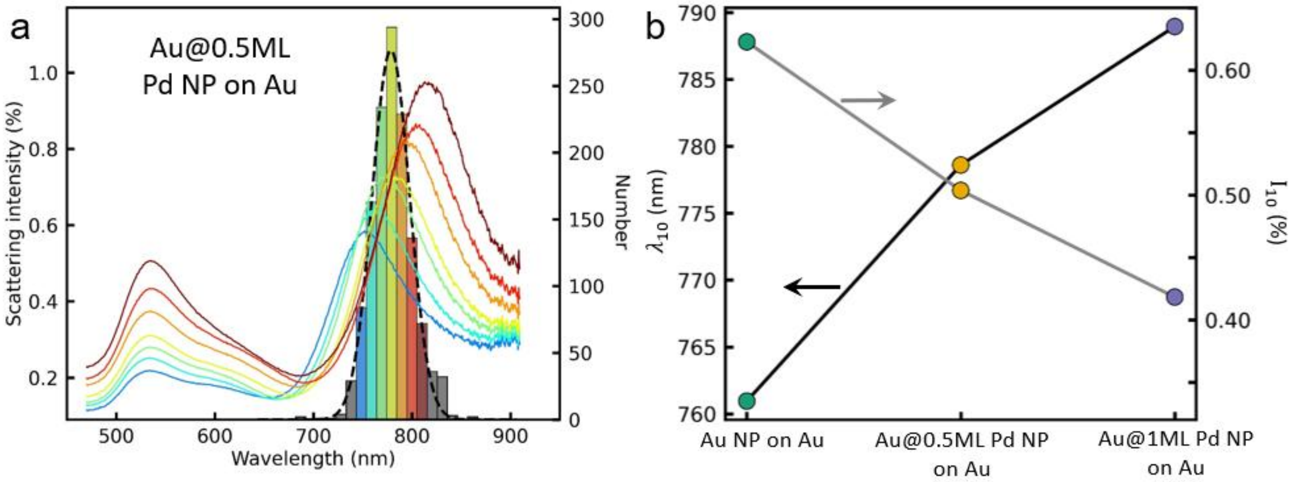

Supplementary Figure 8. a, Histogram of the (10) mode wavelengths and average spectra from each NPoM bin with 0.5 monolayer Pd on Au nanoparticles. **b**, Changes of (10) mode wavelength (λ_{10}) and intensity (I_{10}) for NPoMs with increasing coverage of monolayer Pd on each Au nanoparticle.

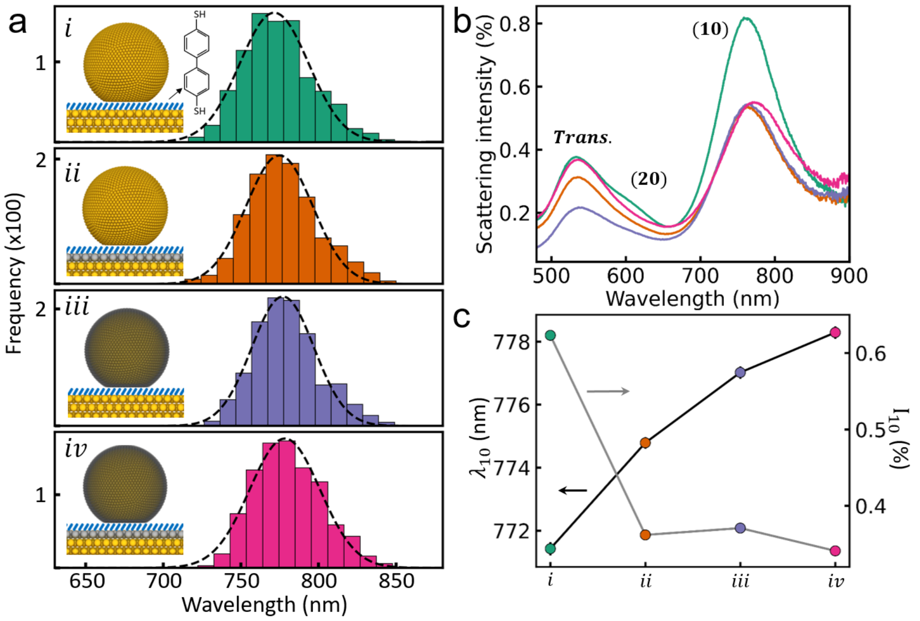

Supplementary Figure 9. **a**, Schematic of NPoMs integrated with a monolayer Pd metal on different sides and their corresponding histogram of (10) mode wavelengths (λ_{10}). The spacer is formed of a SAM of biphenyl dithiol (BPDT) molecules. **b**, Average scattering spectra compared for the most common (modal) bin in (**a**) for the four NPoM geometries. **c**, Intensity (I_0) and resonance wavelength (λ_{10}) compared for the four different geometries in (**a**).

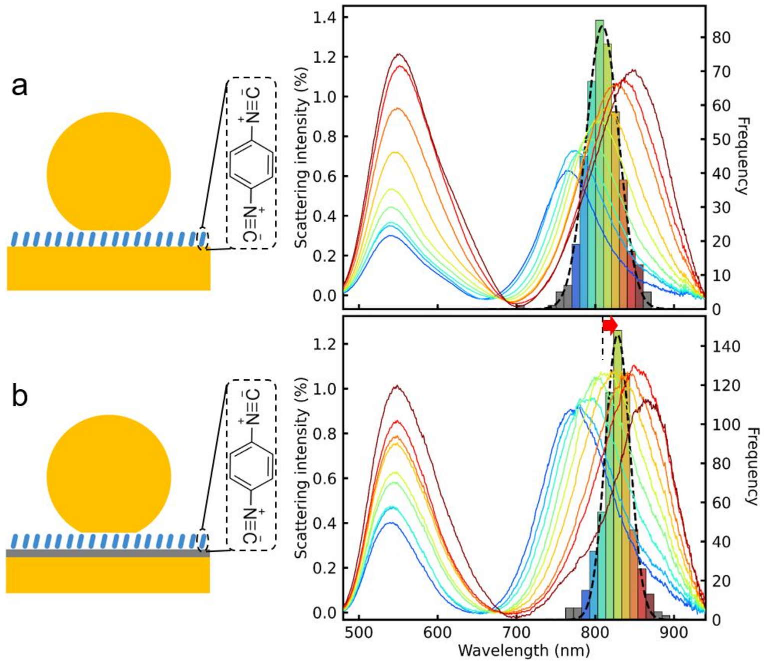

Supplementary Figure 10. Histogram of (10) mode wavelengths and average spectra from each bin of NPoMs (**a**) without and (**b**) with monolayer Pd on the Au film. Spacer is 1,4-phenylene diisocyanide molecular SAM.

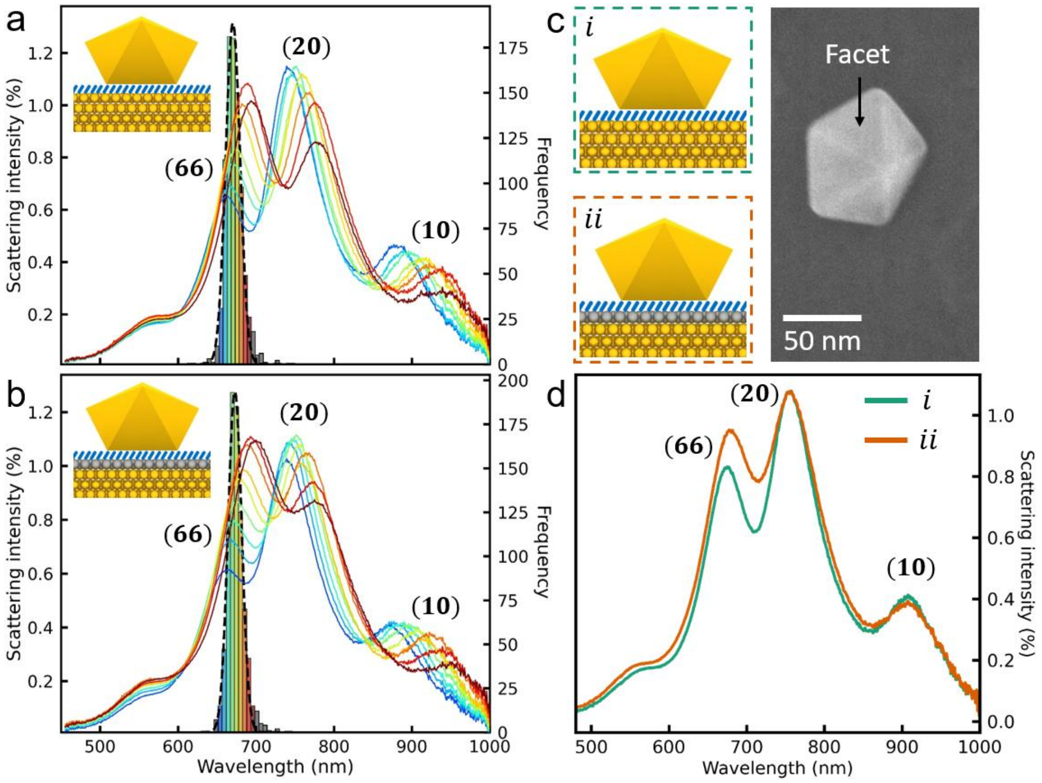

Supplementary Figure 11. Histograms of the (66) mode wavelength and average spectra from each bin of nanodecahedra-on-mirror (NDoM) constructs, (**a**) without and (**b**) with monolayer Pd on the Au film. Spacer is biphenyl thiol molecular SAM. **c**, Schematic (left) and SEM image (right) of NDoM. **d**, Average spectra compared for NDoMs in (**a, b**) atr the most common (modal) bin.

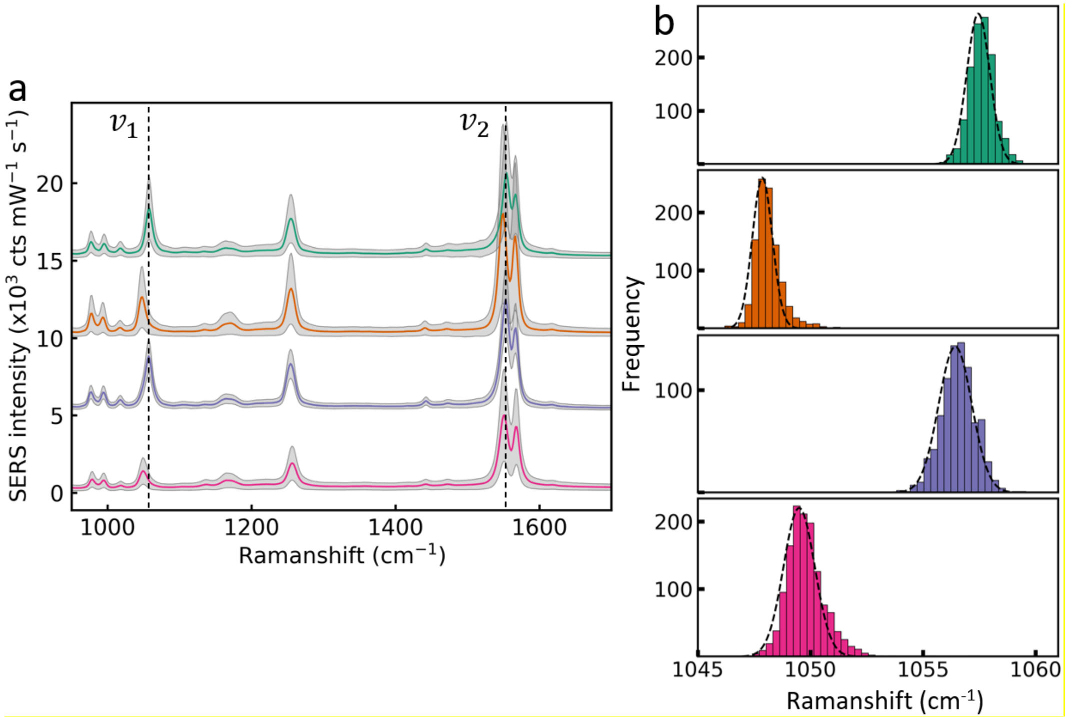

Supplementary Figure 12. a, Average SERS spectra (each over >1000 individual constructs) of different NPoM geometries in Figure 3, with the standard deviation labeled as shaded area (peak area histograms in main text Fig.3e). **b,** Histogram of v_1=1058cm^{-1} frequency distribution for the four different NPoM constructs in (**a**).

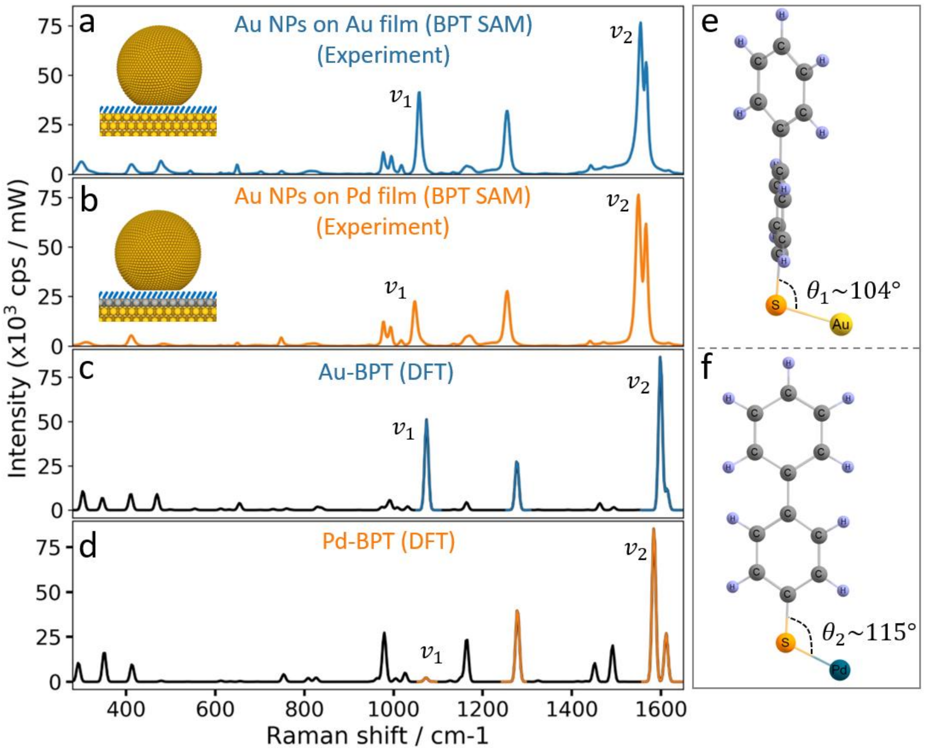

Supplementary Figure 13. Average SERS spectra of NPoMs (**a**) without and (**b**) with monolayer Pd on Au film, together with (**c, d**) their DFT simulated spectra. Spacer is biphenyl thiol molecular SAM. (**e, f**) Optimized molecular structure for simulations in (**c, d**), respectively. Simulation indicates tilt angle differential between BPT on Au and on Pd is ~11°. Average tilt angle of BPT on Au is ~30° according to the literature[3], hence BPT SAM on Pd has estimated tilt of ~19°.

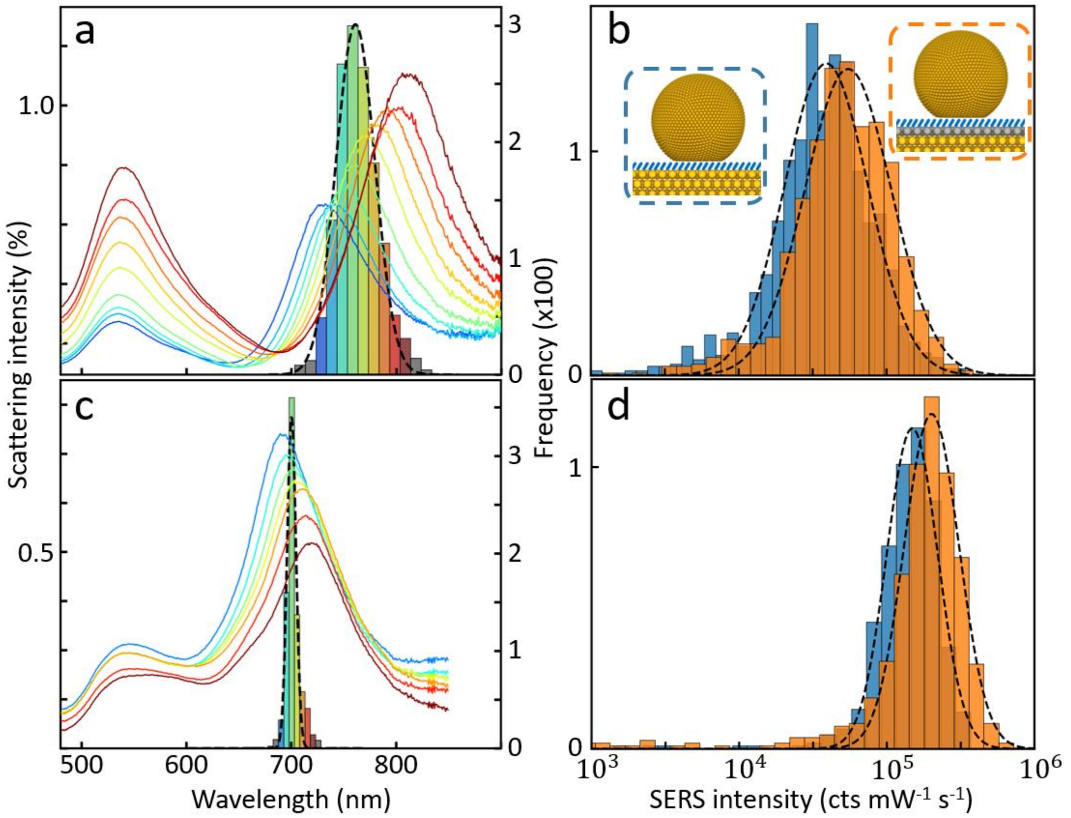

Supplementary Figure 14. (**a, c**) Histograms of the (10) mode wavelength and average spectra from each bin of (**a**) 80nm NPoMs and (**c**) 60nm UPoMs (ultraspherical nanoparticles-on-mirror).[4] (**b, d**) Histograms of ν_2=1554cm^{-1} SERS intensity of individual (**b**) 80nm NPoMs and (**d**) 60nm UPoMs before (blue) and after (orange) Pd-glazing on the bottom facet,

showing same non-resonant chemical enhancement of ~50%. This shows monolayer Pd induced SERS enhancement is not dependent on the (10) resonance wavelength.

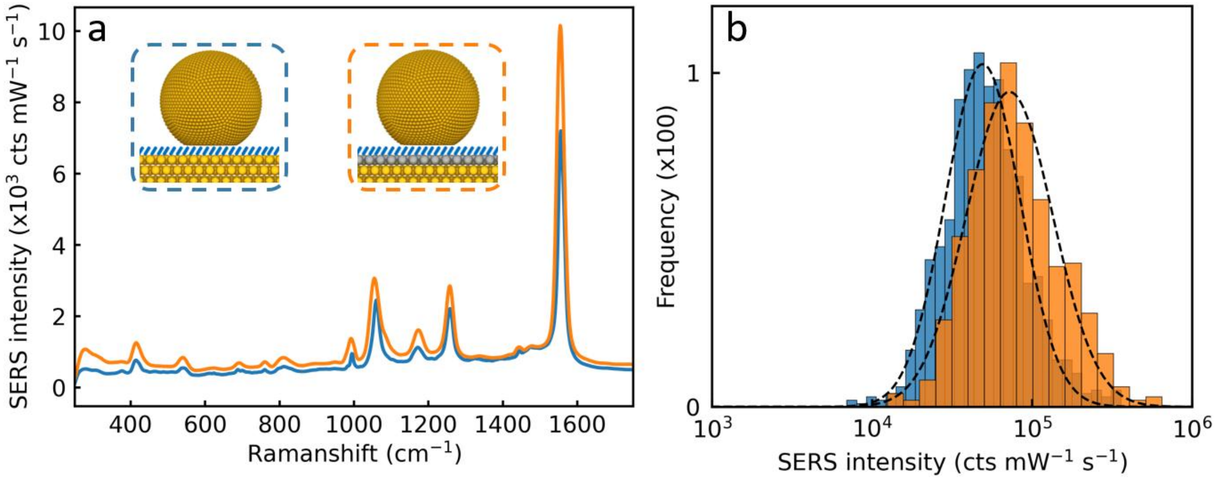

Supplementary Figure 15. a, Average SERS spectra and **b**, histogram of 1556cm^{-1} SERS intensity of > 1600 NPoMs with a SAM of biphenyl dithiol (BPDT). The blue and orange curves are without and with Pd glazing on the bottom facet, showing same ~50% enhancement as BPT SAM.

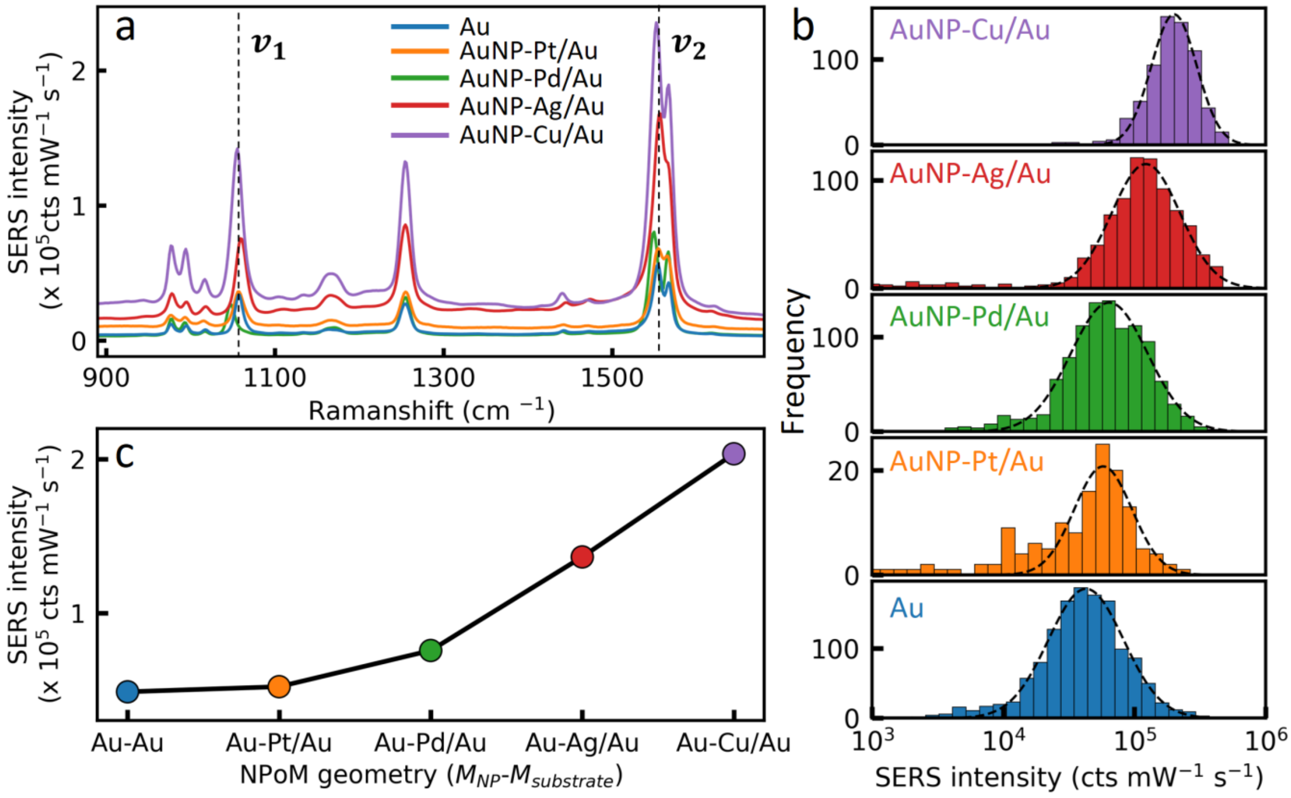

Supplementary Figure 16. (**a**) Average SERS spectra, (**b**) histograms, and (**c**) average of 1556cm^{-1} SERS intensity of 3973 NPoMs with different monolayer metals glazed on their bottom mirrors.

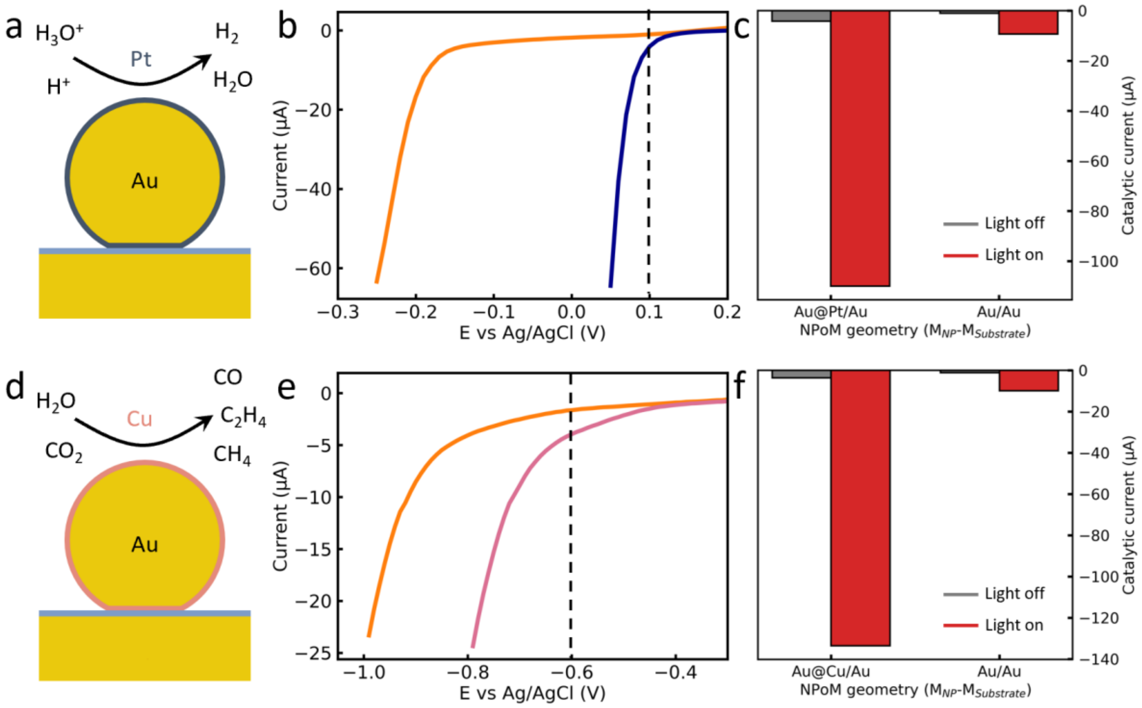

Supplementary Figure 17. (**a, d**) Schematics, (**b, e**) linear sweep voltammetries and (**c, f**) photocatalytic current measurements (the applied potential are 0.1V and -0.6V for Pt and Cu, respectively) of (**a-c**) Pt and (**d-f**) Cu glazed NPoMs in a electrolyte of 0.1 M H_2SO_4 (hydrogen evolution reaction) and 0.1 M $KHCO_3$ (CO_2 reduction reaction). The Pt and Cu glazed NPoM show a catalytic current increase of 26- and 37- fold with the assist of light. The orange curves in (**b, e**) are obtained using Au NPoMs, where the Pt (dark blue) and Cu (purple red) glazed NPoMs show significant reduce in the onset potential of the reactions.

Supplementary Table 1. Summary of glazing monolayer catalytic metal films on bulk Au using UPD deposition.

Metal	Characterization methods	Film quality	General precursor
Au	—	—	—
**Ag**	Electrochemical methods,[5] SEXAFS,[6] X-ray diffraction,[7] STM,[5,8] AFM,[9] EQCM,[10] SHG,[11] and UHV techniques[12]	Continuous	Ag_2SO_4
**Pt**	Electrochemical methods,[13] EQCM,[14] STM,[13] HR-TEM,[15] XPS[14,16]	Partial bilayer island	K_2PtCl_4
**Pd**	Electrochemical methods,[17,18] STM,[17,18] XRD,[19] *in situ* SXRD,[20] HR-TEM,[21] EQCM[22]	Continuous	$PdSO_4$
**Rh**	Electrochemical methods,[23] STM,[23] HR-TEM,[24] AFM,[25] XPS,[26] EQCM[27,28]	Partial bilayer island	$RhCl_3$
**Ir**	Electrochemical methods,[29] STM,[29] HAADF-STEM,[29] STEM-XEDX[29]	Partial nanoislands	K_3IrCl_6
**Cu**	Electrochemical methods,[30,31] STM,[31] AFM,[32] X-ray techniques,[33-35] EQCM,[36] SHG,[11] UHV technique,[37] Operando EC-STEM[38]	Continuous	$CuSO_4$
**Ni**	Electrochemical methods,[39,40] STM,[40,41] XPS,[39,42] EQCM,[42] HR-TEM[42]	Continuous	$NiSO_4$

**Fe**	Electrochemical methods,[43] STM,[43-46] XRD,[44] *in situ* PMOKE,[47] LEED,[48] RHEED[49]	Partial bilayer island	$FeSO_4$
**Ru**	Electrochemical method,[50,51] STM,[50-52] XPS[53]	Partial bilayer island	$RuCl_3$
**Co**	Electrochemical methods,[43,54,55] STM,[43,54-57] AGFM,[58] X-ray scattering,[59] SXRD[60], PMOKE,[61] XRD[61]	Continuous	$CoSO_4$
**Zn**	Electrochemical methods,[62-64] STM,[62,65,66] XANES,[67] Auger electron spectroscopy[66]	Continuous	$Zn(ClO_4)_2$
**Re**	Electrochemical methods,[68,69] XPS,[68] EQCM,[70] HR-TEM[71]	Hydration oxide monolayer ($Re_xO_Y·nH_2O$)	$HReO_4$
**Ti**	Electrochemical methods,[72,73] STM[72,73]	Nanoislands (~1nm)	$TiCl_4$

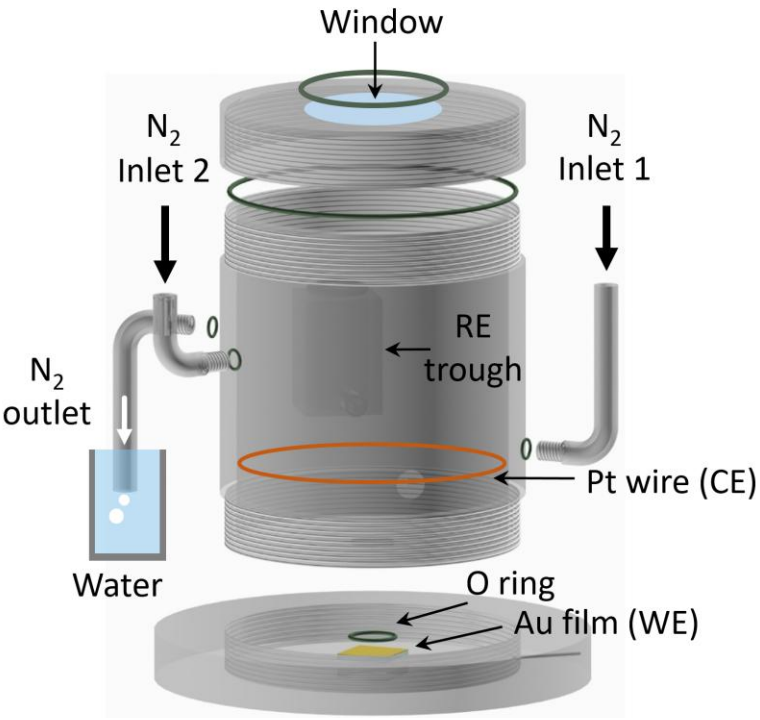

Supplementary Figure 18. Schematic of the electrochemcial cell used for electrochemical deposition and photocatalytic measurements. Inlet 1 pumps N_2 into the electrolyte for de-aeration before measurements, while inlet 2 provides N_2 atmospheric protection during measurements. Outlet is sealed with water to isolate from air. Circular Pt wire is placed on top of working electrode (WE) as a counter electrode (CE) to ensure uniform current distribution. A standard commerical saturated calomel electrode (SCE) is placed in the trough to utilize as a reference electrode (RE).

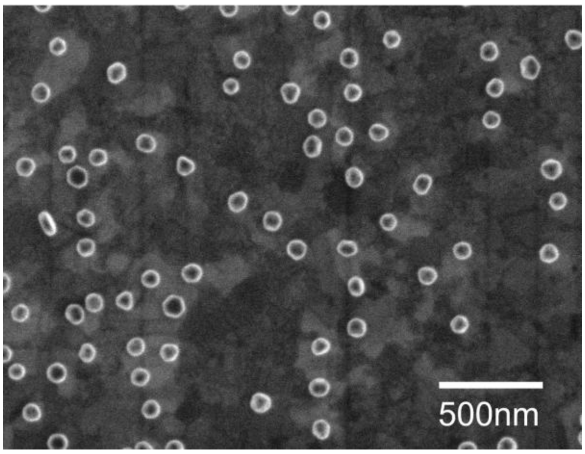

Supplementary Figure 19. SEM image showing density of Au NPoMs used in photocatalytic measurements.

References

1. Zhong J-H*, et al.* Probing the electronic and catalytic properties of a bimetallic surface with 3 nm resolution. *Nat. Nanotechnol.* **12**, 132-136 (2017).
2. Hu J, Hoshi N, Uosaki K, Ikeda K. Vibrational spectroscopic observation of atomic-scale local surface sites using site-selective signal enhancement. *Nano Lett.* **15**, 7982-7986 (2015).
3. Turchanin A, Käfer D, El-Desawy M, Wöll C, Witte G, Gölzhäuser A. Molecular mechanisms of electron-induced cross-linking in aromatic SAMs. *Langmuir* **25**, 7342-7352 (2009).
4. Hu S*, et al.* Full Control of Plasmonic Nanocavities Using Gold Decahedra‐on‐Mirror Constructs with Monodisperse Facets. *Adv. Sci.*, 2207178 (2023).
5. Esplandiu M, Kolb D, Schneeweiss M. An in situ scanning tunneling microscopy study of Ag electrodeposition on Au (111). *Phys. Chem. Chem. Phys.* **1**, 4847-4854 (1999).
6. Samant MG, Borges G, Melroy OR. In situ surface EXAFS study of an underpotentially deposited silver monolayer on gold (111). *J. Electrochem. Soc.* **140**, 421 (1993).
7. Chabala E, Ramadan A, Brunt T, Rayment T. In situ and real time study of an electrode process by differential X-ray diffraction. Part 1. Ag underpotential deposition on Au (111). *J. Electroanal. Chem.* **412**, 67-75 (1996).
8. Garcia S, Salinas D, Mayer C, Schmidt E, Staikov G, Lorenz W. Ag UPD on Au (100) and Au (111). *Electrochim. Acta* **43**, 3007-3019 (1998).
9. Chen CH, Vesecky SM, Gewirth AA. In situ atomic force microscopy of underpotential deposition of silver on gold (111). *J. Am. Chem. Soc.* **114**, 451-458 (1992).
10. Uchida H, Miura M, Watanabe M. Electrochemical quartz crystal microbalance study of silver ad-atoms on highly ordered Au(111) electrodes in sulfuric acid. *J. Electroanal. Chem.* **386**, 261 (1995).
11. Koos D, Richmond G. Structure and stability of underpotentially deposited layers on gold (111) studied by optical second harmonic generation. *J. Phys. Chem* **96**, 3770-3775 (1992).
12. Mrozek P*, et al.* Coadsorption of sulfate anions and silver adatoms on the Au (111) single crystal electrode. Ex situ and in situ comparison. *Electrochim. Acta* **40**, 17-28 (1995).
13. Brankovic S, Wang J, Adžić R. Metal monolayer deposition by replacement of metal adlayers on electrode surfaces. *Surf. Sci.* **474**, L173-L179 (2001).
14. Liu Y, Gokcen D, Bertocci U, Moffat TP. Self-terminating growth of platinum films by electrochemical deposition. *Science* **338**, 1327-1330 (2012).
15. Hong W, Li CW. Microstructural Evolution of Au@ Pt Core–Shell Nanoparticles under Electrochemical Polarization. *ACS Appl. Mater. Interfaces* **11**, 30977-30986 (2019).
16. Li M, Ma Q, Zi W, Liu X, Zhu X, Liu S. Pt monolayer coating on complex network substrate with high catalytic activity for the hydrogen evolution reaction. *Sci. Adv.* **1**, e1400268 (2015).
17. Kibler L, Kleinert M, Randler R, Kolb D. Initial stages of Pd deposition on Au (hkl) Part I: Pd on Au (111). *Surf. Sci.* **443**, 19-30 (1999).
18. Kibler L, Kleinert M, Kolb D. Initial stages of Pd deposition on Au (hkl): Part II: Pd on Au (100). *Surf. Sci.* **461**, 155-167 (2000).

19. Naohara H, Ye S, Uosaki K. Epitaxial growth of a palladium layer on an Au (100) electrode. *J. Electroanal. Chem.* **473**, 2-9 (1999).
20. Sibert E, Wang L, De Santis M, Soldo-Olivier Y. Mechanisms of the initial steps in the Pd electro-deposition onto Au (111). *Electrochim. Acta* **135**, 594-603 (2014).
21. Ding Y, Fan F, Tian Z, Wang ZL. Atomic structure of Au− Pd bimetallic alloyed nanoparticles. *J. Am. Chem. Soc.* **132**, 12480-12486 (2010).
22. Naohara H, Ye S, Uosaki K. Electrochemical layer-by-layer growth of palladium on an Au (111) electrode surface: Evidence for important role of adsorbed Pd complex. *J. Phys. Chem. B* **102**, 4366-4373 (1998).
23. Kibler L, Kleinert M, Kolb D. The initial stages of rhodium deposition on Au (111). *J. Electroanal. Chem.* **467**, 249-257 (1999).
24. Chantry RL, Siriwatcharapiboon W, Horswell SL, Logsdail AJ, Johnston RL, Li Z. Overgrowth of rhodium on gold nanorods. *J. Phys. Chem. C.* **116**, 10312-10317 (2012).
25. Smiljanić M, Srejić I, Grgur B, Rakočević Z, Štrbac S. Hydrogen evolution on Au (111) catalyzed by rhodium nanoislands. *Electrochem. commun.* **28**, 37-39 (2013).
26. Štrbac S, Smiljanić M, Rakočević Z. Spontaneously deposited Rh on Au (111) observed by AFM and XPS: Electrocatalysis of hydrogen evolution. *J. Electrochem. Soc.* **163**, D3027 (2016).
27. Langerock S, Heerman L. Study of the electrodeposition of rhodium on polycrystalline gold electrodes by quartz microbalance and voltammetric techniques. *J. Electrochem. Soc.* **151**, C155 (2004).
28. Mech K, Żabiński P, Kowalik R. Analysis of rhodium electrodeposition from chloride solutions. *J. Electrochem. Soc.* **161**, D458 (2014).
29. Ahn SH, Tan H, Haensch M, Liu Y, Bendersky LA, Moffat TP. Self-terminated electrodeposition of iridium electrocatalysts. *Energy Environ. Sci.* **8**, 3557-3562 (2015).
30. Schultze J, Dickertmann D. Potentiodynamic desorption spectra of metallic monolayers of Cu, Bi, Pb, Tl, and Sb adsorbed at (111),(100), and (110) planes of gold electrodes. *Surf. Sci.* **54**, 489-505 (1976).
31. Hachiya T, Honbo H, Itaya K. Detailed underpotential deposition of copper on gold (III) in aqueous solutions. *J. electroanal. chem. interfacial electrochem.* **315**, 275-291 (1991).
32. Manne S, Hansma P, Massie J, Elings V, Gewirth A. Atomic-resolution electrochemistry with the atomic force microscope: copper deposition on gold. *Science* **251**, 183-186 (1991).
33. Toney MF*, et al.* Electrochemical deposition of copper on a gold electrode in sulfuric acid: resolution of the interfacial structure. *Phys. Rev. Lett.* **75**, 4472 (1995).
34. Melroy O*, et al.* In-plane structure of underpotentially deposited copper on gold (111) determined by surface EXAFS. *Langmuir* **4**, 728-732 (1988).
35. Abruna H, Gog T, Materlik G, Uelhoff W. X-Ray standing wave study of copper underpotentially deposited on Au (100). *J. Electroanal. Chem.* **360**, 315-323 (1993).
36. Watanabe M, Uchida H, Miura M, Ikeda N. Electrochemical quartz crystal microbalance study of copper ad-atoms on highly ordered Au (111) electrodes in sulfuric acid. *J. Electroanal. Chem.* **384**, 191-195 (1995).
37. Zhang J, Sung YE, Rikvold PA, Wieckowski A. Underpotential deposition of Cu on Au (111) in sulfate-containing electrolytes: A theoretical and experimental study. *J. Chem. Phys.* **104**, 5699-5712 (1996).
38. Yang Y, Shao Y-T, DiSalvo FJ, Muller DA, Abruña HD. Metal monolayers on command: underpotential deposition at nanocrystal surfaces: a quantitative operando electrochemical transmission electron microscopy study. *ACS Energy Lett.* **7**, 1292-1297 (2022).
39. Zafeiratos S, Paloukis FE, Neophytides SG. Nickel electrodeposition on a gold polycrystalline foil: a combined voltammetric and photoelectron spectroscopy study. *J. Phys. Chem. B* **108**, 1371-1379 (2004).
40. Möller F, Kintrup J, Lachenwitzer A, Magnussen O, Behm R. In situ STM study of the electrodeposition and anodic dissolution of ultrathin epitaxial Ni films on Au (111). *Phys. Rev. B* **56**, 12506 (1997).
41. Möller F, Magnussen O, Behm R. Two-dimensional needle growth of electrodeposited Ni on reconstructed Au (111). *Phys. Rev. Lett.* **77**, 3165 (1996).
42. Wang R, Bertocci U, Tan H, Bendersky LA, Moffat TP. Self-terminated electrodeposition of Ni, Co, and Fe ultrathin films. *J. Phys. Chem. C.* **120**, 16228-16237 (2016).
43. Gündel A, Cagnon L, Gomes C, Morrone A, Schmidt J, Allongue P. In-situ magnetic measurements of electrodeposited ultrathin Co, Ni and Fe/Au (111) layers. *Phys. Chem. Chem. Phys.* **3**, 3330-3335 (2001).
44. Jurca H*, et al.* Epitaxial electrodeposition of Fe on Au (111): structure, nucleation, and growth mechanisms. *J. Phys. Chem. C.* **120**, 16080-16089 (2016).
45. Voigtländer B, Meyer G, Amer NM. Epitaxial growth of Fe on Au (111): a scanning tunneling microscopy investigation. *Surf. Sci. Lett.* **255**, L529-L535 (1991).
46. Donati F, Mairov A, Casari CS, Passoni M, Bassi AL. Nucleation and growth mechanisms of Fe on Au (111) in the sub-monolayer regime. *Surf. Sci.* **606**, 702-710 (2012).

47. Gündel A, Devolder T, Chappert C, Schmidt J, Cortes R, Allongue P. Electrodeposition of Fe/Au (1 1 1) ultrathin layers with perpendicular magnetic anisotropy. *Physica B: Condensed Matter* **354**, 282-285 (2004).
48. Allmers T, Donath M. Growth and morphology of thin Fe films on flat and vicinal Au (111): a comparative study. *New J. Phys.* **11**, 103049 (2009).
49. Dekadjevi D, Hickey B, Brown S, Hase T, Fulthorpe B, Tanner B. Structural phase transition of Fe grown on Au (111). *Phys. Rev. B* **71**, 054108 (2005).
50. Strbac S, Maroun F, Magnussen OM, Behm RJ. The structure, growth and reactivity of electrodeposited Ru/Au (111) surfaces. *J. Electroanal. Chem.* **500**, 479-490 (2001).
51. Thambidurai C, Kim Y-G, Stickney JL. Electrodeposition of Ru by atomic layer deposition (ALD). *Electrochim. Acta* **53**, 6157-6164 (2008).
52. Strbac S, Magnussen O, Behm R. Nanoscale pattern formation during electrodeposition: Ru on reconstructed Au (111). *Phys. Rev. Lett.* **83**, 3246 (1999).
53. Rettew RE, Guthrie JW, Alamgir FM. Layer-by-layer Pt growth on polycrystalline Au: surface-limited redox replacement of overpotentially deposited Ni monolayers. *J. Electrochem. Soc.* **156**, D513 (2009).
54. Cagnon L*, et al.* Enhanced interface perpendicular magnetic anisotropy in electrodeposited Co/Au (111) layers. *Phys. Rev. B* **63**, 104419 (2001).
55. Allongue P, Cagnon L, Gomes C, Gündel A, Costa V. Electrodeposition of Co and Ni/Au (1 1 1) ultrathin layers. Part I: nucleation and growth mechanisms from in situ STM. *Surf. Sci.* **557**, 41-56 (2004).
56. Di N, Kubal J, Zeng Z, Greeley J, Maroun F, Allongue P. Influence of controlled surface oxidation on the magnetic anisotropy of Co ultrathin films. *Appl. Phys. Lett.* **106**, (2015).
57. Di N, Damian A, Maroun F, Allongue P. Influence of potential on the electrodeposition of co on Au (111) by in situ stm and reflectivity measurements. *J. Electrochem. Soc.* **163**, D3062 (2016).
58. Allongue P, Maroun F, Jurca HF, Tournerie N, Savidand G, Cortès R. Magnetism of electrodeposited ultrathin layers: Challenges and opportunities. *Surf. Sci.* **603**, 1831-1840 (2009).
59. Reikowski F*, et al.* In situ surface X-ray diffraction study of ultrathin epitaxial Co films on Au (111) in alkaline solution. *Electrochim. Acta* **197**, 273-281 (2016).
60. Lucas CA*, et al.* Film and interface atomic structures of electrodeposited Co/Au (111) layers: an in situ X-ray scattering study as a function of the surface chemistry and the electrochemical potential. *J. Phys. Chem. C.* **120**, 3360-3370 (2016).
61. Prod'homme P*, et al.* Preparation, characterization and magneto-optical investigations of electrodeposited Co/Au films. *J. Magn. Magn. Mater.* **315**, 26-38 (2007).
62. Dogel J, Freyland W. Layer-by-layer growth of zinc during electrodeposition on Au (111) from a room temperature molten salt. *Phys. Chem. Chem. Phys.* **5**, 2484-2487 (2003).
63. Quaiyyum MA, Aramata A, Moniwa S, Taguchi S, Enyo M. Underpotential deposition of Zn2+ ions on platinum, palladium and gold at various pH values. *J. Electroanal. Chem.* **373**, 61-66 (1994).
64. Taguchi S, Kondo M, Mori H, Aramata A. Formation of zinc–oxianion complex adlayer by underpotential deposition of Zn on Au (1 1 1) electrode: Preferential formation of zinc monohydrogen phosphate complex in weakly acidic solutions. *Electrochim. Acta* **111**, 642-655 (2013).
65. Nakamura M, Aramata A, Yamagishi A, Taniguchi M. Underpotential deposition of zinc ions on Au (111) in phosphate solution (pH 4.6): kinetic and STM studies. *J. Electroanal. Chem.* **446**, 227-231 (1998).
66. Schuett FM, Heubach MK, Mayer J, Ceblin MU, Kibler LA, Jacob T. Electrodeposition of zinc onto Au (111) and Au (100) from the ionic liquid [MPPip][TFSI]. *Angew. Chem., Int. Ed.* **133**, 20624-20631 (2021).
67. Lee JR, O'Malley RL, O'Connell TJ, Vollmer A, Rayment T. X-ray absorption spectroscopy characterization of Zn underpotential deposition on Au (1 1 1) from phosphate supporting electrolyte. *Electrochim. Acta* **55**, 8532-8538 (2010).
68. Rivera J, Garcia-Garcia R, Coutino-Gonzalez E, Orozco G. Electrochemical study in acid aqueous solution and ex-situ X-ray photoelectron spectroscopy analysis of metallic rhenium surface. *J. Electroanal. Chem.* **893**, 115297 (2021).
69. Szabó S, Bakos I. Electroreduction of rhenium from sulfuric acid solutions of perrhenic acid. *J. Electroanal. Chem.* **492**, 103-111 (2000).
70. Schrebler R*, et al.* Study of the electrodeposition of rhenium thin films by electrochemical quartz microbalance and X-ray photoelectron spectroscopy. *Thin Solid Films* **483**, 50-59 (2005).
71. Vargas-Uscategui A, Mosquera E, Cifuentes L. Analysis of the electrodeposition process of rhenium and rhenium oxides in alkaline aqueous electrolyte. *Electrochim. Acta* **109**, 283-290 (2013).
72. Endres F*, et al.* On the electrodeposition of titanium in ionic liquids. *Phys. Chem. Chem. Phys.* **10**, 2189-2199 (2008).
73. Mukhopadhyay I, Aravinda C, Borissov D, Freyland W. Electrodeposition of Ti from TiCl4 in the ionic liquid l-methyl-3-butyl-imidazolium bis (trifluoro methyl sulfone) imide at room temperature: study on phase formation by in situ electrochemical scanning tunneling microscopy. *Electrochim. Acta* **50**, 1275-1281 (2005).
